# Low‐dose testosterone administration and oestrogen synthase availability in the female brain: A pilot study

**DOI:** 10.1111/jne.70154

**Published:** 2026-03-02

**Authors:** Manon Dubol, My Jonasson, Kayo Takahashi, Johan Wikström, Yasuyoshi Watanabe, Gunnar Antoni, Mark Lubberink, Anat Biegon, Inger Sundström‐Poromaa, Erika Comasco

**Affiliations:** ^1^ Department of Women's and Children's Health, Science for Life Laboratory Uppsala University Uppsala Sweden; ^2^ Department of Surgical Sciences, Nuclear Medicine and PET Uppsala University Uppsala Sweden; ^3^ RIKEN Center for Biosystems Dynamics Research Kobe Japan; ^4^ Department of Surgical Sciences, Neuroradiology Uppsala University Uppsala Sweden; ^5^ Department of Medicinal Chemistry Uppsala University Uppsala Sweden; ^6^ Department of Radiology and Neurology Stony Brook University School of Medicine Stony Brook New York USA; ^7^ Department of Women's and Children's Health Uppsala University Uppsala Sweden

**Keywords:** brain, hypoactive sexual desire disorder, oestrogen synthase, testosterone, women's health

## Abstract

Testosterone and oestrogens play significant roles in female physiology, extending beyond reproductive functions to influence brain health, mood regulation, and behaviour. Testosterone low‐dose therapy is increasingly considered for alleviating sexual dysfunction symptoms in postmenopausal women, and has been recently investigated as therapy for depressive symptoms, though the mechanisms and safety of this approach are not entirely clear. Specifically, the effects of testosterone use on brain oestrogen synthase (aromatase), which maintains the balance between androgens and oestrogens, remain unexplored. This study investigated the effects of short‐term, low‐dose testosterone administration on brain oestrogen synthase availability and associated mood and behavioural changes in healthy women. Healthy women were exposed to 1 week of low‐dose testosterone (10 mg/day). Binding of oestrogen synthase was examined by [^11^C]cetrozole positron emission tomography before and during testosterone exposure. Psychometric assessment of depression, anxiety, and aggression was performed at the same time. Peripheral testosterone levels were significantly increased (up to 33‐fold) upon treatment, which had no significant effect on brain oestrogen synthase binding in the thalamus, as supported by Bayesian analyses, nor in the hypothalamus and amygdala. Psychometric measures of depression, anxiety, and aggression also remained unchanged by testosterone treatment. These findings suggest that short‐term, clinically relevant testosterone administration has no major effects on the brain oestrogen synthase availability in healthy women, which may reassure patients with hypoactive sexual desire disorder considering this treatment. Larger, long‐term studies are needed to confirm these results and explore effects in patients with clinical need for testosterone treatment.

## 1 INTRODUCTION

Testosterone and oestrogens exert an extended role in female physiology beyond traditional reproductive functions, impacting brain health, mood regulation, and sexual function.^1^, ^2^ Oestrogen synthase (also called aromatase) catalyses the conversion of androgens to oestrogens, thus playing a central role in maintaining neuroendocrine balance. In the human brain, oestrogen synthase is primarily found in the thalamus, amygdala, and hypothalamus,^3^, ^4^ hence in place to influence emotion processing, aggression, cognitive function, mood, and sexual drive.^5^

Testosterone and other androgens have historically been studied predominantly in men, largely due to their higher circulating levels and more widespread clinical and recreational use, resulting in a substantial literature on androgen‐related effects on mood disorders and aggression.^6^ Much less is known about the effects of androgens on the female brain, although therapeutic low doses of testosterone are used in women, especially in cases of low sexual desire, hypogonadism, or menopausal symptom management.^7^ The prevalence of hypoactive sexual desire disorder (HSDD) as defined in the Diagnostic and Statistical Manual of Mental Disorders, Fifth Edition (DSM‐5)^8^ is 8 to 19% in women across Europe and the United States.^9^ In addition, with the worldwide increasing numbers of post‐menopausal women and related adverse health consequences, sexual dysfunction and the associated distress might impact about a billion individuals by 2030.^7^ The International Society for the Study of Women's Sexual Health Clinical Practice Guideline indicates the use of systemic transdermal testosterone as treatment for HSDD in postmenopausal and late reproductive premenopausal women.^7^ Furthermore, testosterone has been investigated as therapy for depression in adult women.^10^, ^11^, ^12^, ^13^, ^14^, ^15^

In women with sex steroid deficiency, transdermal testosterone treatment also improves psychological well‐being and depressive symptoms.^12^, ^13^ Besides, using a sublingual 0.5 mg single dose of testosterone in young healthy women, van Honk and colleagues observed a reduced performance in cognitive empathy measured by the Reading the Mind in the Eyes Test,^16^ though criticism on the task warrants cautious interpretation.^17^ Side effects of testosterone in women are most commonly reversible and include hirsutism and acne. Yet the impact of testosterone therapy on women's brains remains understudied, despite its widespread off‐label application.^18^

**FIGURE 1:**
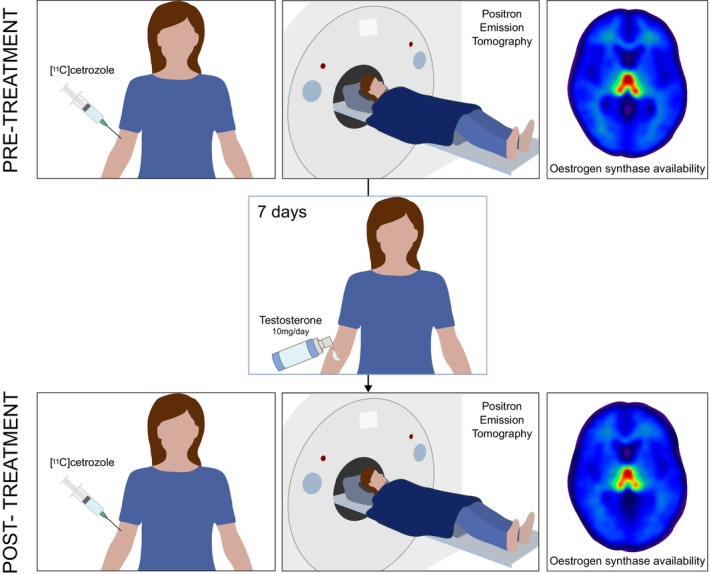
Study design. Each participant underwent twice 90‐min PET imaging with the [^11^C]cetrozole radiotracer targeting oestrogen synthase, once at baseline (pre‐treatment) and once following a 1‐week transdermal treatment with testosterone (post‐treatment). The two sessions were scheduled at least two months apart. Axial slices (*z* = 2, MNI coordinates) of [^11^C]cetrozole non‐displaceable binding potential (BP_ND_) parametric maps, averaged across all participants (*n* = 10), show the highest aromatase availability in the thalamus.

Understanding whether testosterone administration can alter oestrogen synthase in the brain of women is especially relevant in assessing the neuroendocrine effects of testosterone treatment and implications for therapeutic strategies.^19^ Several animal studies have demonstrated an upregulation of oestrogen synthase expression and activity following exposure to high levels of androgens both in vivo and in vitro,^20^, ^21^ which led us to hypothesize that testosterone administration may result in upregulated oestrogen synthase levels in the brain. Specifically, preclinical studies have shown that androgen exposure increased oestrogen synthase mRNA and protein expression, enhanced enzymatic activity in hypothalamic and limbic regions, and increased in vivo binding of radiolabelled oestrogen synthase inhibitors, with effects demonstrated predominantly in males but also present in females, in a region‐ and context‐dependent manner (e.g., gonadal status, dose, and duration).^20^, ^21^ Notably, in male rodents, testosterone has been shown to increase oestrogen synthase activity within 24–48 h after treatment.^22^ Moreover, rapid, non‐genomic modulation of its enzymatic activity may also occur within minutes through phosphorylation‐dependent mechanisms.^21^ Thus, this pilot study sought to investigate the effects of short‐term, clinically relevant, low‐dose testosterone administration on brain oestrogen synthase availability in healthy women assessed with [^11^C]cetrozole positron emission tomography (PET), a tracer with high sensitivity and specificity,^23^ and putative associated behavioural changes (Figure 1).

## 2 METHODS

The present investigation is part of the Brain Sex Hormones (BSH) project, which focuses on the effects of both endogenous and exogenous sex hormones on brain function and behaviour in healthy women. The study was conducted at the Department of Women's and Children's Health at Uppsala University Hospital; all procedures were approved by the Regional Ethical Review Board in Uppsala (n. 2014/393), and all participants provided written informed consent after receiving both oral and written explanations about the study.

### 2.1 Participants

Participants were evaluated at the Department of Women's and Children's Health, Uppsala University Hospital. Ten healthy women (ages 22–33) with regular menstrual cycles participated. Exclusion criteria were: pregnancy (current or within the past 6 months), breastfeeding, use of hormonal contraceptives, any current treatment with hormones or psychoactive drugs, nicotine use, chronic neurological conditions, psychiatric disorders, high blood pressure (≥140/90), obesity (BMI ≥30), left‐handedness, and contraindications for MRI and PET scanning. Psychiatric disorders were ruled out using the MINI Neuropsychiatric Interview,^24^ and the absence of pregnancy was confirmed at the start of the scanning session using a pregnancy test. Participants were also asked to follow certain guidelines prior to the sessions, such as fasting for 2 h, avoiding medications and caffeine for 12 h, and ensuring adequate sleep the night before.

### 2.2 Procedures

Two neuroimaging sessions were conducted, one at baseline (prior to treatment initiation) and the other on the last day of a one‐week transdermal testosterone treatment. Participants were instructed and reminded to start applying the testosterone gel exactly 7 days before the scheduled scan. The two PET sessions were conducted at least two menstrual cycles apart to minimize potential carryover effects and ensure hormonal recovery between sessions. Given the very limited (one study on humans showing no effect) and inconsistent evidence (in rodents vs. baboons) on menstrual cycle effects on brain aromatase,^25^, ^26^, ^27^ the two PET scans were scheduled during the same menstrual cycle phase whenever feasible to minimize potential variability related to hormonal fluctuations. A one‐week regimen was chosen, as daily application produces stable circulating concentrations within 2–3 days^28^, ^29^ and elicits central neural effects within hours,^30^, ^31^, ^32^, ^33^ providing several days of sustained steady‐state exposure while limiting cumulative androgen burden for safety.^7^, ^14^ Participants were instructed to apply one press of the testosterone gel (Tostrex 2% Gel®) canister piston, delivering 0.5 g of gel containing 10 mg testosterone, once a day and preferably in the morning. The dose is twice as high as typically prescribed in postmenopausal women, but was chosen to ensure elevated and stable testosterone levels.^34^ The application area varied every day between the abdomen (lower belly area, alternating left and right sides) and the inner thigh (half the dose over an area of at least 10 × 15 cm on each thigh).

In order to link changes in oestrogen synthase availability with changes in mood and behaviour, all participants were asked to fill out the following psychometric questionnaires at the time of recruitment and during both PET scanning sessions: the self‐rating version of the Montgomery‐Åsberg Depression Rating Scale (MADRS‐S),^35^ the State Anxiety Inventory (STAI‐S),^36^ and the Revised Swedish Version of the Aggression Questionnaire (AQ‐RSV).^37^

Venous blood samples were collected at the beginning of both sessions to measure circulating sex steroid levels. Quantification of oestradiol, progesterone, and testosterone concentrations was performed by liquid chromatography–tandem mass spectrometry (LC–MS/MS) at the University of Bergen's Core Facility for Metabolomics. Serum samples (300 μL) underwent protein precipitation with acetonitrile followed by liquid–liquid extraction using ethyl acetate‐heptane. Steroid separation was performed on a C‐18 column using an Acquity UPLC system coupled to a Waters Xevo TQ‐S tandem mass spectrometer with electrospray ionization. Oestradiol was analysed in negative ion mode and progesterone in positive ion mode using multiple reaction monitoring, with two transitions monitored per analyte. The lower limits of quantification were 3.6 pmol/L for oestradiol and 0.21 nmol/L for progesterone. Menstrual cycle phase was determined based on both forward and backward counting using the date of previous menstrual bleeding onset, onset of the next menses when available (otherwise, the participants' self‐reported average menstrual cycle length was used), and confirmed by serum concentrations of progesterone and oestradiol. A summary of menstrual cycle timing and gonadal hormone levels for each PET session is provided in Table S1.

### 2.3 Neuroimaging data acquisition and analysis

Brain oestrogen synthase availability was assessed as previously described by Jonasson, et al.^4^ Briefly, dynamic PET scans lasting 90 min (26 frames of increasing duration) were collected using [^11^C]cetrozole, a radiolabelled oestrogen synthase inhibitor. [^11^C]cetrozole was synthesized following Takahashi et al.^23^ with minor modifications, by reacting [^11^C]methyl iodide with the boronic ester precursor MD‐298, purified by high‐performance liquid chromatography, reformulated in ethanol/Kleptose hydroxypropyl‐β‐cyclodextrin/phosphate buffer, sterilized by filtration, and characterized for radiochemical purity, identity, concentration, and palladium content. Participants received 4 MBq kg‐1 of [^11^C]cetrozole, to a maximum of 400 MBq. The PET scans were conducted using three models of scanners (Discovery ST, Discovery IQ, or Discovery MI PET/CT scanner, GE Healthcare, Milwaukee, WI, USA), with efforts to maintain similar spatial resolution across scanners. Structural MRI scans were performed on a 3 T Achieva dStream scanner (Philips Healthcare, Best, The Netherlands) for anatomical reference and region‐of‐interest (ROI) identification. The dynamic PET images were corrected for motion and co‐registered with MRI scans using the VOIager software (GE Healthcare, Uppsala, Sweden) and SPM8 (Wellcome Trust Center for Neuroimaging, University College London, UK). Brain parcels were defined using PVElab,^38^ with the exception of the left and right amygdala that were manually defined on the [^11^C]cetrozole parametric maps using a 70% isocontour mask. Non‐displaceable binding potential (BP_ND_) was calculated using the Logan reference tissue model (LRTM),^39^ as the distribution volume (VT) ratio between the target and reference regions minus one (DVR‐1), using the cerebellum as reference region.

### 2.4 Statistical analysis

While statistical power is a critical consideration in study design, PET imaging imposes substantial economic and logistical constraints that limit achievable sample sizes. Given these concerns, researchers have increasingly adopted sequential Bayes factor testing as a means to determine when enough data have been collected to reach a conclusion, allowing studies to stop early without inflating error rates.^40^ This approach balances ethical considerations, budget constraints, and scientific rigour. Following this approach, we present data based on 10 participants (Figure S1), a number that is consistent with many exploratory PET studies, particularly those using repeated‐measures designs, where meaningful within‐subject changes in tracer binding can often be detected with modest sample sizes, given the substantially reduced variance.^41^, ^42^ Between‐sessions differences in testosterone concentration and psychometric scores were assessed using paired‐samples *t*‐tests. Similarly, comparisons of menstrual cycle day at scanning and oestradiol and progesterone serum levels between sessions were carried out using Wilcoxon signed‐rank tests. Based on previous reports of [^11^C]cetrozole specific binding in humans and non‐human primates,^23^ ROI‐based analyses focused on brain regions with BP_ND_ ≥ 0.3, including the thalamus, hypothalamus, and amygdala. Within‐subject comparisons of regional BP_ND_ values between the baseline and testosterone sessions were conducted using Wilcoxon signed rank tests, adapted to non‐normally distributed data. Statistical analyses were conducted in Statistical Package for the Social Sciences (SPSS for Windows, version 28). The significance threshold was set at *q* < 0.1, corrected for multiple testing using the False Discovery Rate (FDR). All Bayesian analyses were conducted using JASP (version 0.19.3). Sequential analyses were conducted in JASP to assess how the Bayes Factor evolved as individual participant data were progressively added, providing insight into the stability and accumulation of evidence for the hypothesized treatment effect on oestrogen synthase availability (Figure S1). Bayesian paired‐sample *t*‐tests were used to evaluate the hypothesized increase in testosterone levels, [^11^C]cetrozole BP_ND_, and aggression scores, as well as the hypothesized decrease in depression and anxiety scores, following treatment. One‐tailed (directional) hypotheses were specified accordingly, and the default Cauchy prior width of 0.707 was applied.

## 3 RESULTS

### 3.1 Sample characteristics

Ten women aged 26 ± 4 (mean ± SD) years, with regular menstrual cycles and a healthy body mass index (22.6 ± 2.4) were included. On average, the participants had 15 years in school (post‐secondary education level), seven were university students, and three were working full‐time. The majority were nulliparous, except for one who had given birth. Based on self‐reports, the severity of depressive symptoms, as assessed by the MADRS‐S, was minimal (4.9 ± 1.8), and the STAI‐S scores indicated non‐clinically relevant levels of anxiety (33.2 ± 4.0). Baseline testosterone levels were within the physiological range of young women (0.94 ± 0.38 nmol/L),^43^, ^44^ and the baseline mean AQ‐RSV aggression score was low (18.8 ± 5.9). None of the participants reported side effects during the study.

### 3.2 Short‐term exposure to testosterone

**FIGURE 2:**
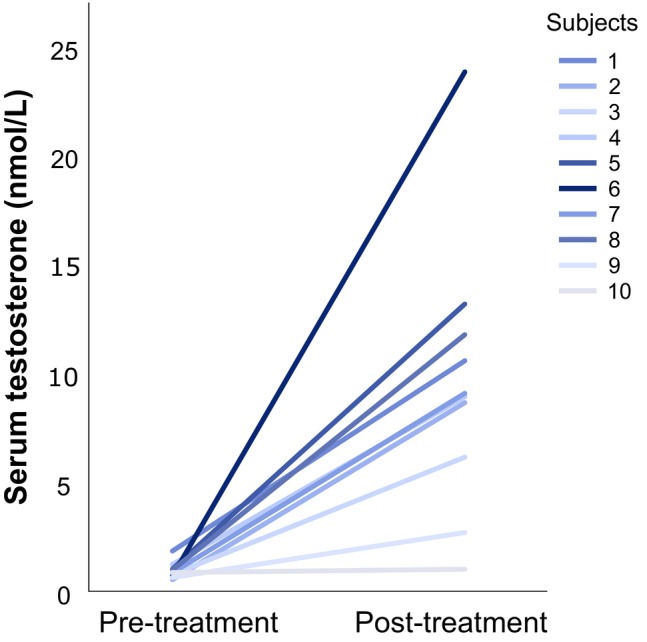
Increase in peripheral testosterone levels upon 1 week of low‐dose testosterone administration. Line graph displaying the individual change in serum concentration of testosterone from the pre‐treatment PET session to the post‐treatment PET session following 1 week of transdermal testosterone treatment, for the 10 participants included in the study. The individual increase in testosterone concentration is colour‐coded, with light grey reflecting the minimal increase and dark blue indicating the maximal increase observed in the study sample. The increase in systemic testosterone levels ranged from 18 to 3329%.

**FIGURE 3:**
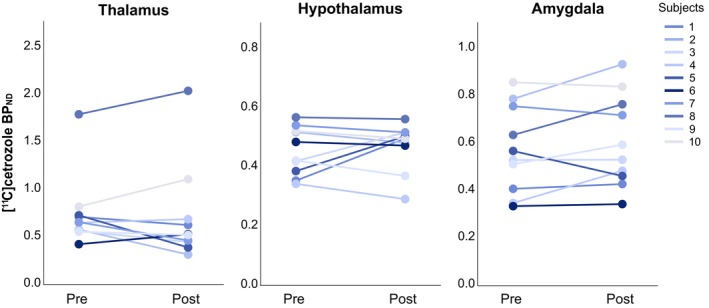
Effect of low‐dose testosterone administration on oestrogen synthase availability. Line graphs displaying the individual change in [^11^C]cetrozole non‐displaceable binding potential (BP_ND_) estimates in regions‐of‐interest, from the pre‐treatment PET session (Pre) to the post‐treatment PET session (Post) following 1 week of transdermal testosterone treatment. The colour‐coding represents the individual increase in testosterone concentration, with light grey reflecting the minimal increase and dark blue indicating the maximal increase observed in the study sample. Across regions, no consistent pattern of change in oestrogen synthase availability appeared in the study group, in relation to the change in testosterone levels.

Following low‐dose testosterone administration, testosterone levels increased between 18% and up to 3329% (Figure 2). Between the two sessions, the average serum concentration of testosterone increased ninefold, with a mean increase of 8.65 nmol/L (treatment average concentration of 9.59 ± 6.29 nmol/L; *t* = −4.36, *p* = .002, Cohen's *d* = 1.38). Menstrual cycle day at scanning and serum oestradiol and progesterone levels did not differ between the baseline and the treatment sessions (Table S1). No statistically significant testosterone‐induced change in brain oestrogen synthase availability was detected as measured by [^11^C]cetrozole BP_ND_ (Figure 3). Due to non‐normality of the PET data, the median and interquartile range (IQR) are reported below, as the primary measure of central tendency. Treatment levels of oestrogen synthase were similar to baseline values, for the thalamus (median_pre_ = 0.66, IQR_pre_ = 0.19; median_post_ = 0.53, IQR_post_ = 0.36, *W* = 22, *p* = .57, *r*_rank‐biserial_ = 0.6), hypothalamus (median_pre_ = 0.45, IQR_pre_ = 0.15; median_post_ = 0.49, IQR_post_ = 0.07, *W* = 27, *p* = .96, *r*_rank‐biserial_ = 0.5), and amygdala (median_pre_ = 0.55, IQR_pre_ = 0.37; median_post_ = 0.56, IQR_post_ = 0.33, *W* = 40, *p* = .20, *r*_rank‐biserial_ = 0.3). In line with this, serum testosterone levels were not associated with oestrogen synthase availability during treatment, nor was the change in testosterone levels associated with the change in oestrogen synthase availability (Tables S2 and S3). Similarly, depression symptoms, anxiety symptoms, and level of aggression remained unchanged with treatment (Table 1; Figure S2). Neither serum testosterone levels nor the change in testosterone levels were associated with psychometrics at the treatment assessment (Tables S4 and S5).

**TABLE 1:** Effect of low‐dose testosterone administration on aggression, depression, and anxiety.

	Pre‐treatment	Post‐treatment	*t*‐value	*p*‐value	Cohen's *d*
AQ‐RSV	19.67 (6.56)	18.3 (7.00)	0.48	.65	0.20
MADRS‐S	6.70 (7.42)	9.60 (8.43)	−1.17	.27	−0.37
STAI‐S	37.60 (11.39)	36.30 (8.63)	0.48	.64	0.15

*Note*: Changes in aggression scores (AQ‐RSV), depression symptom severity (MADRS‐S), and anxiety symptom severity (STAI‐S) were explored using Student *t*‐tests. Mean (standard deviation) are presented.

Abbreviations: AQ‐RSV, Aggression Questionnaire—Revised Swedish Version; MADRS‐S, Montgomery‐Åsberg Depression Rating Scale—Self rating version; STAI‐S, State–Trait Anxiety Inventory—State form.

The Bayesian analyses provided strong evidence in favour of the expected increase in testosterone serum levels following treatment (BF_10_ = 49.42; Figure S3), indicating good participant compliance with the study protocol. In contrast, there was moderate evidence supporting the null hypothesis (i.e., no change) for [^11^C]cetrozole binding potential in the thalamus (BF_10_ = 0.22; Figure S4) and for both depression (BF_10_ = 0.16) and aggression (BF_10_ = 0.28) scores (Figure S5). As to changes in anxiety levels and [^11^C]cetrozole binding potential in the hypothalamus, the evidence pointing to the null hypothesis was anecdotal (BF_10_ = 0.45 and BF_10_ = 0.52, respectively; Figures S4 and S5), as was the evidence for increases in [^11^C]cetrozole binding in the amygdala (BF_10_ = 1.13; Figure S4).

## 4 DISCUSSION

The present findings describe the effects of one week of transdermal testosterone treatment at a clinically relevant dose on brain oestrogen synthesis and associated mood and aggressiveness in healthy women. Short‐term testosterone administration has been shown to modulate neural activity in emotion‐ and threat‐processing circuits within hours to days of dosing^30^, ^32^, ^33^ supporting the suitability of a 7‐day regimen for assessing potential central effects on oestrogen synthase availability within the limbic brain. Testosterone administration did not result in any significant changes in brain oestrogen synthase availability, as indicated by consistent availability of oestrogen synthase at the baseline and treatment assessments. Similarly, no substantial change was observed at the behavioural level.

To date, no study has investigated the impact of testosterone treatment on oestrogen synthase availability in the female human brain. Preclinical studies indicate that oestrogen synthase mRNA, protein and activity levels are upregulated by androgens.^21^ As detailed by Cornil,^21^ this androgenic regulation has been demonstrated across species using several complementary approaches including in situ hybridization, immunohistochemistry, and enzyme activity assays, demonstrating that testosterone increases oestrogen synthase expression and activity within hypothalamic and limbic regions. These convergent findings are further supported by in vivo PET studies, which have shown that the use of anabolic‐androgenic steroids increases oestrogen synthase availability in rats^23^, ^45^, ^46^, ^47^ and rhesus macaques,^46^ whereas the antiandrogen flutamide has the opposite effect.^47^ Notably, those in vivo studies used male specimens exclusively, with a dose of androgen considerably higher than the dose employed in our study, which is similar (and even slightly higher) to the dose used clinically in women. Whether the lack of effect of low‐dose, short‐term treatment with testosterone reported here reflects a species difference, a sex difference, or a dose effect is not entirely clear, especially given the often‐contradictory reports on the psychosocial^48^ and neural^49^ effects of high‐dose, long‐term testosterone use in female‐to‐male transgender individuals.

In this context, it is maybe not surprising that, in the current sample of healthy women, testosterone administration had no significant effect on measures of depression, anxiety and aggression levels. While psychometric outcomes are discussed here, the primary aim of this study was to investigate the neurobiological effects of short‐term testosterone administration on oestrogen synthase availability; any mood‐ or aggression‐related findings should thus be interpreted as secondary and hypothesis‐generating rather than evidence of clinical efficacy. Comparable data on the short‐term effect of low‐dose testosterone administration on mood in healthy premenopausal women is scarce. While a few studies pointed to a beneficial effect on mood after months of transdermal testosterone use,^10^, ^11^, ^12^, ^13^ the global consensus on the use of testosterone therapy for women is that the available data do not show an effect on depressed mood or general wellbeing.^14^ This is further supported by the negative results of a recent randomized placebo‐controlled study conducted in women with antidepressant‐resistant depression.^15^ Extensive research highlights the modulatory role of testosterone on reactive aggression, involving enhanced amygdala reactivity and reduced prefrontal–amygdala coupling during the processing of social threat cues; but evidence on human aggression is weak.^50^ Particularly, the effect of daily testosterone use on human aggression remains unclear. Studies on the long‐term use of testosterone at a low dose in late reproductive premenopausal women,^51^ and high doses in transgender men^52^ point to no change in aggressiveness. In sum, while the evidence on mental health and behavioural side effects of testosterone use in women is scarce, the present findings align with previous reports of unaltered mood and aggressiveness following low‐dose testosterone treatment.

The absence of significant oestrogen synthase modulation in response to low‐dose testosterone treatment could have implications for the use of testosterone therapies in clinical populations. While the neuroendocrine mechanisms by which testosterone influences sexual desire in women are not fully understood, it remains debated whether these effects are mediated via direct androgen receptor activation or through aromatization to oestradiol and subsequent oestrogen receptor signalling. Importantly, accumulating evidence in humans suggests that testosterone's beneficial effects on female sexuality may primarily be androgen receptor‐mediated and do not require conversion to oestradiol.^7^ In this context, the present findings provide reassurance for women undergoing low‐dose testosterone therapy for conditions such as low sexual desire, hypogonadism, or menopausal symptom management, that such treatment is unlikely to disrupt local oestrogen production within the brain and related processes.^53^ However, these conclusions are drawn from a specific population of healthy, normo‐weight females, and generalization to other groups, particularly those with different genetic, hormonal or metabolic profiles, warrants caution. It is also possible that transient or regionally confined changes in oestrogen synthase activity, below the detection threshold of PET imaging or occurring at a functional rather than expression level, contribute to behavioural effects.

Notably, assessing testosterone levels in vivo in the human brain is currently not achievable. While oestrogen synthase availability does not directly mirror local concentrations of testosterone or oestrogen, it represents the enzymatic capacity for converting androgens to oestrogens, thereby influencing the local androgen–oestrogen balance. Experimental manipulations of oestrogen synthase activity have been shown to produce opposite changes in testosterone and oestrogen levels, supporting its role as a functional regulator of neurosteroid dynamics.^54^ Importantly, the regional distribution of oestrogen synthase availability measured with [^11^C]cetrozole PET is highly consistent with that revealed by post‐mortem and molecular studies,^5^ supporting the validity of [^11^C]cetrozole binding as a proxy for oestrogen synthase expression in vivo. Of note, the PET tracer [^11^C]cetrozole measuring oestrogen synthase in the brain has a higher signal‐to‐noise ratio, greater selectivity and specificity, and improved metabolic stability compared to its predecessor, [^11^C]vorozole.^23^ PET further offers the unique advantage of enabling longitudinal within‐subject assessment of enzymatic availability, complementing insights from invasive and post‐mortem methods. Oestrogen synthase availability in the brain could thus provide insights into the neuroendocrine regulation of behaviour and brain function.^3^, ^4^

Methodological considerations when interpreting the findings of the present study include the short duration of testosterone administration, which may not reflect effects of long‐term exposure or responses to varying doses. Nevertheless, previous studies have reported rapid modulation of central neural circuits within hours to days of testosterone dosing,^30^, ^32^, ^33^ suggesting that our 1‐week design was adequate to capture potential short‐term effects. Supporting this rationale, animal studies have shown that testosterone administration can significantly increase hypothalamic oestrogen synthase activity within 24–48 h, with maximal effects observed after about 1 week in male rats,^22^ indicating that short‐term exposure may be sufficient to induce enzymatic changes, although longer durations could reveal additional effects. It should be noted, on the other hand that the rapid, non‐genomic membrane‐initiated actions of neurosteroids occurring within seconds or minutes are mechanistically distinct from the gene‐ or protein‐level regulation of aromatase assessed with PET.^55^ No association has been found between menstrual cycle phase and oestrogen synthase expression in the human brain,^25^ and the results remained virtually the same when controlling for menstrual cycle phase. Thus, it is unlikely that the variability, albeit minute, in the endogenous testosterone levels within the participants assessed at different time points of the menstrual cycle has an influence on central processes.^56^ Additionally, while the final sample included 10 participants (as supported by sequential Bayesian testing of the change in thalamic [^11^C]cetrozole binding; Figure S1), this number is consistent with many published PET studies in the field (for instance, references 57, 58), where sample sizes are often constrained by the complexity, cost, and logistical demands of PET imaging. Meaningful within‐subject changes in radiotracer binding can often be detected with modest sample sizes (e.g., 8–12 participants), as the variance is substantially reduced. It is however important to acknowledge that subtle effects could not be reliably detected, which may limit the generalizability of our findings. Consequently, while no significant treatment effect was observed, Bayesian analyses indicated that the evidence in favour of the null hypothesis was only anecdotal for some outcomes, suggesting that the absence of effect should be interpreted with caution. The present study is therefore positioned as a pilot investigation aimed at identifying any substantial short‐term effects of low‐dose testosterone treatment on brain oestrogen synthase availability and associated psychological functioning. Future studies incorporating measures of tissue‐level oestrogen synthase expression would be valuable to corroborate the present findings and further elucidate the mechanisms underlying central oestrogen synthase regulation. Thus, although preliminary, the study provides important initial insights into the effects of short‐term testosterone treatment on oestrogen synthase availability in the brain, highlighting the need for further research with larger samples to confirm and extend these findings, and explore variability across different populations of patients receiving testosterone as treatment. In line with this, studies involving individuals with hormonal dysregulation, or different menopausal stages, or gender dysphoria, could offer insights into whether brain oestrogen synthase responds differently in those with altered baseline steroid environments.

In conclusion, the present study provides preliminary evidence that low‐dose, short‐term testosterone administration does not significantly alter brain oestrogen synthase levels in healthy women. This mechanistic finding suggests that such therapy, under appropriate clinical guidelines, may have limited impact on brain‐derived oestrogen synthesis. Behavioural outcomes, specifically measures of mood and aggression, showed no significant change. However, these secondary analyses were exploratory and should be interpreted as hypothesis‐generating rather than evidence of clinical efficacy. Future research should aim to elucidate the neurobiological mechanisms underlying the effects of testosterone, particularly in brain regions relevant to cognitive and emotional processing, as well as neuroplasticity, where oestrogen synthase can act as a modulator. Longitudinal studies involving varied dosages and populations are essential to fully understand the implications of testosterone therapy and its potential benefits or risks in clinical contexts.

## AUTHOR CONTRIBUTIONS

**Manon Dubol:** Formal analysis; data visualization; writing – original draft, review and editing. **My Jonasson:** Data curation (PET); writing – review and editing. **Kayo Takahashi:** Methodology (PET); validation; writing – review and editing. **Johan Wikström:** Methodology; investigation (MR); writing – review and editing. **Yasuyoshi Watanabe:** Methodology (PET); validation; writing – review and editing. **Gunnar Antoni:** Methodology; investigation (PET); writing – review and editing. **Mark Lubberink:** Data curation (PET); writing – review and editing. **Anat Biegon:** Supervision; writing – review and editing. **Inger** **Sundström‐Poromaa:** Conceptualization; investigation; funding acquisition; writing – review and editing; project administration; resources; supervision. **Erika Comasco:** Conceptualization; investigation; funding acquisition; writing – review and editing; project administration; resources; supervision.

## CONFLICT OF INTEREST STATEMENT

The authors declare no conflicts of interest.

## Supporting information


**Figure S1.** Sequential analysis of the Bayes Factor for the effect of testosterone treatment on thalamic aromatase availability. The sequential analysis plot shows how the Bayes Factor for the hypothesized increase in [^11^C]cetrozole binding potential (BF_10_) evolves as each participant's data is added to the analysis. The blue area illustrates the evidence for the alternative hypothesis (H_1_), which posits that aromatase availability increases after one‐week low‐dose transdermal testosterone treatment. The grey area illustrates the evidence for the null hypothesis (H_0_), which posits that aromatase availability does not change after one‐week low‐dose transdermal testosterone treatment. In the thalamus, where the highest aromatase availability is found, evidence gradually strengthens in favour of H_0_ as the sample accumulates, reaching moderately strong evidence (BF_10_ = 0.22) by the final observations (*n* = 10). The pie chart depicts the posterior probability mass favouring H_0_ over H_1_.
**Figure S2.** Effect of low‐dose testosterone administration on psychometrics. Bar graphs of (A) MADRS‐S, (B) STAI‐S and (C) AQ‐RSV scores collected during scanning sessions, before (pre‐treatment) and after one‐week of transdermal testosterone treatment (post‐treatment). The error bars represent 2× standard error of the mean.
**Figure S3.** Robustness check of the Bayes Factor for the effect of testosterone treatment on testosterone serum levels. The robustness plot shows how the Bayes Factor (BF_10_) for the increase in serum testosterone changes with different Cauchy prior widths. The blue area illustrates the evidence for the alternative hypothesis (H_1_), which posits that serum testosterone levels increase after one‐week low‐dose transdermal testosterone treatment. The grey area illustrates the evidence for the null hypothesis (H_0_), which posits that central testosterone levels do not change after one‐week low‐dose transdermal testosterone treatment. Across a wide range of priors (default user prior: *r* = 0.707; wide prior: *r* = 1; ultrawide prior: *r* = 1.414), the evidence remains very strong in favour of the alternative hypothesis (H_1_). The red dot indicates the maximum BF_10_ obtained (54.61 at *r* = 1.184), demonstrating that the result is robust to reasonable variations in the prior specification.
**Figure S4.** Robustness check of the Bayes Factor for the effect of testosterone treatment on aromatase availability. The robustness plots show how the Bayes Factor (BF_10_) for the increase in [^11^C]cetrozole binding changes with different Cauchy prior widths. The blue areas illustrate the evidence for the alternative hypothesis (H_1_), which posits that aromatase availability increases after one‐week low‐dose transdermal testosterone treatment. The grey areas illustrate the evidence for the null hypothesis (H_0_), which posits that aromatase availability does not change after one‐week low‐dose transdermal testosterone treatment. Across a wide range of priors (default user prior: *r* = 0.707; wide prior: *r* = 1; ultrawide prior: *r* = 1.414), the evidence supports H_0_, for the thalamus. BF_10_ values indicate that the strength of the evidence is moderate for the absence of changes in aromatase availability in the thalamus, weak (anecdotal) for the absence of changes in aromatase availability in the hypothalamus, and weak (anecdotal) for the increase in aromatase availability in the amygdala.
**Figure S5.** Robustness check of the Bayes Factor for the effect of testosterone treatment on psychometrics. The robustness plots show how the Bayes Factor (BF_10_) for the decrease in depression (MADRS) and anxiety (STAI‐S) scores, as well as for the increase in self‐rated aggression (AQ‐RSV), with different Cauchy prior widths. The blue areas illustrate the evidence for the alternative hypothesis (H_1_), which posits that changes in mood and behaviour are seen after one‐week low‐dose transdermal testosterone treatment, as described above. The grey areas illustrate the evidence for the null hypothesis (H_0_), which posits that psychometric scores do not change after one‐week low‐dose transdermal testosterone treatment. Across a wide range of priors (default user prior: *r* = 0.707; wide prior: *r* = 1; ultrawide prior: *r* = 1.414), the evidence supports H_0_ for depression and aggression scores. BF_10_ values indicate that the strength of the evidence is moderate for the absence of changes in MADRS and AQ‐RSV scores, and weak (anecdotal) for the absence of changes in STAI‐S scores.
**Table S1.** Menstrual cycle timing and hormonal characteristics across the baseline and post‐treatment sessions. Between‐sessions differences in menstrual cycle day at scanning (based on forward counting), and serum gonadal hormones were assessed using paired‐samples Wilcoxon signed rank tests in SPSS for Windows, version 30. Most participants were assessed in the early follicular phase of the menstrual cycle.
**Table S2.** Association between the post‐treatment peripheral testosterone levels and the post‐treatment oestrogen synthase availability. Spearman rho (*ρ*) and *p* values are presented, illustrating the relationship between the serum testosterone concentrations (T) and the [^11^C]cetrozole binding (BP_ND_) following one‐week of transdermal testosterone treatment.
**Table S3.** Association between the pre‐ to post‐treatment change in peripheral testosterone levels and the change in oestrogen synthase availability. Spearman rho (ρ) and *p* values are presented, illustrating the relationship between the change in serum testosterone concentrations (T) and the change in [^11^C]cetrozole binding (BP_ND_) from the pre‐treatment to the post‐treatment scanning session.
**Table S4.** Association between the post‐treatment peripheral testosterone levels and the post‐treatment psychometrics. Pearson coefficient (*r*) and *p* values are presented, illustrating the relationship between the serum testosterone concentrations (T) and the depression (MADRS), anxiety (STAI‐S) and aggression (AQ‐RSV) scores following one‐week of transdermal testosterone treatment.
**Table S5.** Association between the pre‐ to post‐treatment change in peripheral testosterone levels and the change in psychometrics. Pearson coefficient (*r*) and *p* values are presented, illustrating the relationship between the change in serum testosterone concentrations (T) and the change in depression (MADRS), anxiety (STAI‐S) and aggression (AQ‐RSV) scores from the pre‐treatment to the post‐treatment scanning session.

## Data Availability

The data that support the findings of this study are available from the corresponding author upon reasonable request.
