# Supplementary material for: Low‐dose testosterone administration and oestrogen synthase availability in the female brain: A pilot study
Source: J Neuroendocrinol. 2026 Mar 2;38(3):e70154. doi: 10.1111/jne.70154 (PMC12952942; doi:10.1111/jne.70154)
Supplement: Supplementary file 1 — Figure S1. Sequential analysis of the Bayes Factor for the effect of testosterone treatment on thalamic aromatase availability. The sequential analysis plot shows how the Bayes Factor for the hypothesized increase in [11C]cetrozole binding potential (BF10) evolves as each participant's data is added to the analysis. The blue area illustrates the evidence for the alternative hypothesis (H1), which posits that aromatase availability increases after one‐week low‐dose transdermal testosterone treatment. The grey area illustrates the evidence for the null hypothesis (H0), which posits that aromatase availability does not change after one‐week low‐dose transdermal testosterone treatment. In the thalamus, where the highest aromatase availability is found, evidence gradually strengthens in favour of H0 as the sample accumulates, reaching moderately strong evidence (BF10 = 0.22) by the final observations (n = 10). The pie chart depicts the posterior probability mass favouring H0 over H1. Figure S2. Effect of low‐dose testosterone administration on psychometrics. Bar graphs of (A) MADRS‐S, (B) STAI‐S and (C) AQ‐RSV scores collected during scanning sessions, before (pre‐treatment) and after one‐week of transdermal testosterone treatment (post‐treatment). The error bars represent 2× standard error of the mean. Figure S3. Robustness check of the Bayes Factor for the effect of testosterone treatment on testosterone serum levels. The robustness plot shows how the Bayes Factor (BF10) for the increase in serum testosterone changes with different Cauchy prior widths. The blue area illustrates the evidence for the alternative hypothesis (H1), which posits that serum testosterone levels increase after one‐week low‐dose transdermal testosterone treatment. The grey area illustrates the evidence for the null hypothesis (H0), which posits that central testosterone levels do not change after one‐week low‐dose transdermal testosterone treatment. Across a wide range of priors (default user pr [file JNE-38-e70154-s001.docx]

**
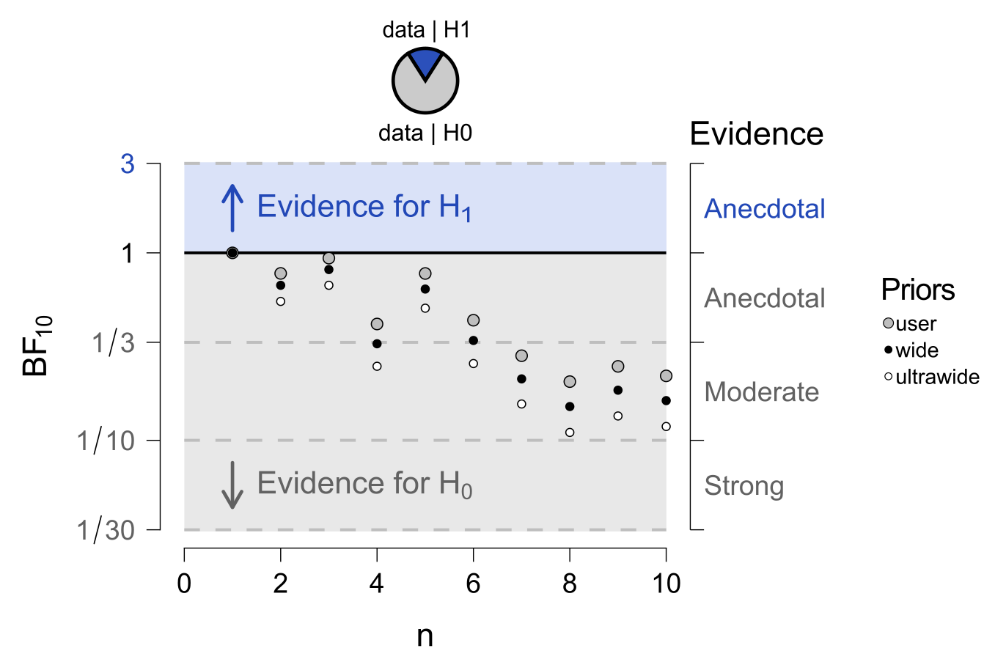
Supplement to: Low-dose testosterone administration and oestrogen synthase availability in the female brain**

**Figure S1. Sequential analysis of the Bayes Factor for the effect of testosterone treatment on thalamic aromatase availability.** The sequential analysis plot shows how the Bayes Factor for the hypothesized increase in [^11^C]cetrozole binding potential (BF_10_) evolves as each participant’s data is added to the analysis. The blue area illustrates the evidence for the alternative hypothesis (H_1_), which posits that aromatase availability increases after one-week low-dose transdermal testosterone treatment. The grey area illustrates the evidence for the null hypothesis (H_0_), which posits that aromatase availability does not change after one-week low-dose transdermal testosterone treatment. In the thalamus, where the highest aromatase availability is found, evidence gradually strengthens in favour of H_0_ as the sample accumulates, reaching moderately strong evidence (BF_10_ = 0.22) by the final observations (n=10). The pie chart depicts the posterior probability mass favouring H_0_ over H_1_.


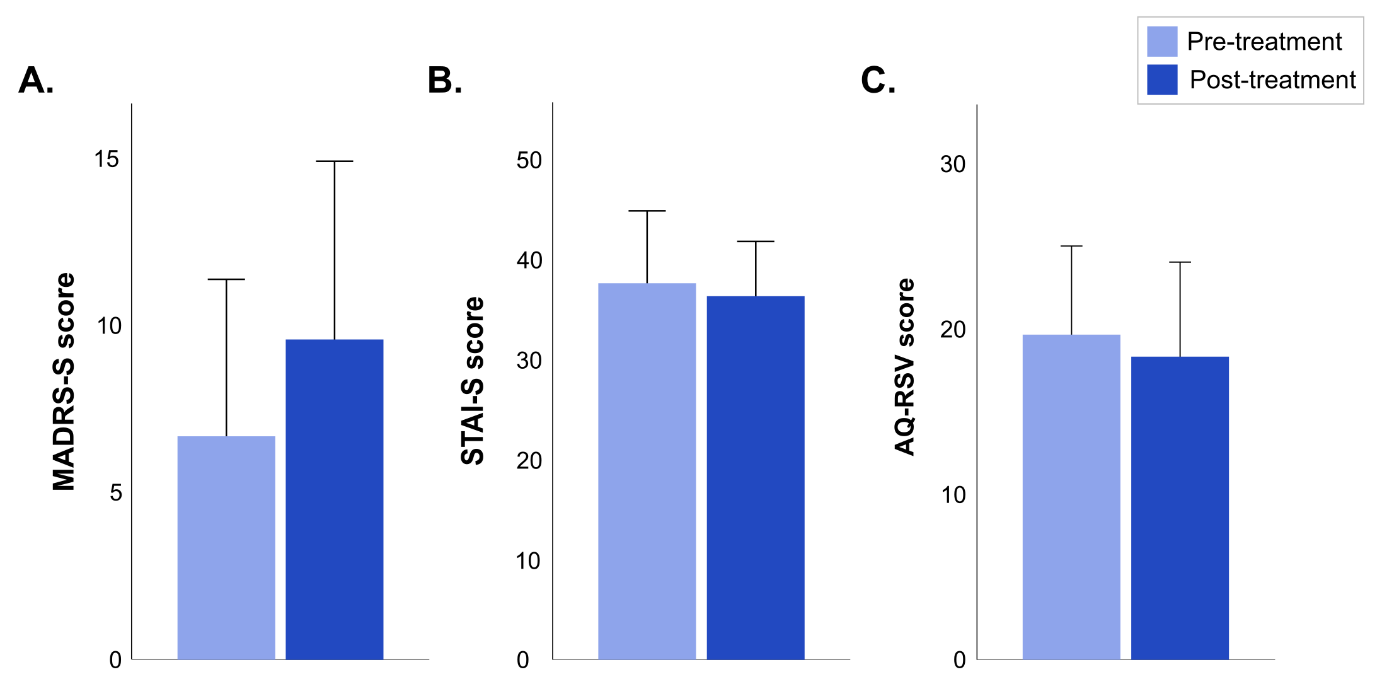


**Figure S2. Effect of low-dose testosterone administration on psychometrics.** Bar graphs of **A.** MADRS-S, **B.** STAI-S and **C.** AQ-RSV scores collected during scanning sessions, before (pre-treatment) and after one-week of transdermal testosterone treatment (post-treatment). The error bars represent 2x standard error of the mean.

**
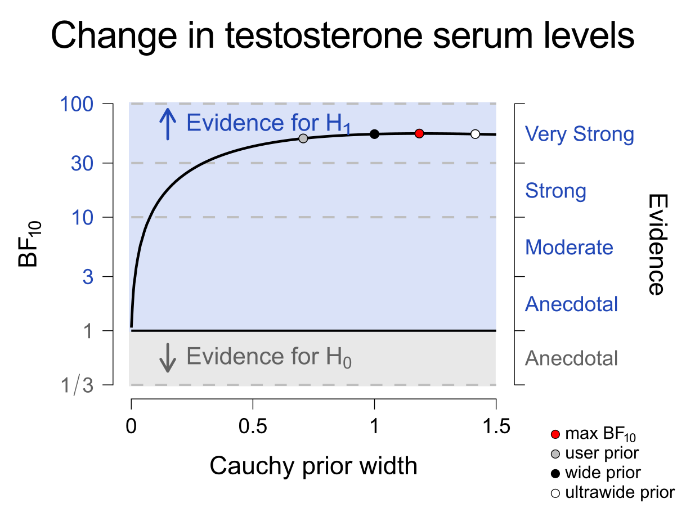
**

**Figure S3. Robustness check of the Bayes Factor for the effect of testosterone treatment on testosterone serum levels.** The robustness plot shows how the Bayes Factor (BF_10_) for the increase in serum testosterone changes with different Cauchy prior widths. The blue area illustrates the evidence for the alternative hypothesis (H_1_), which posits that serum testosterone levels increase after one-week low-dose transdermal testosterone treatment. The grey area illustrates the evidence for the null hypothesis (H_0_), which posits that central testosterone levels do not change after one-week low-dose transdermal testosterone treatment. Across a wide range of priors (default user prior: r = 0.707; wide prior: r = 1; ultrawide prior: r = 1.414), the evidence remains very strong in favour of the alternative hypothesis (H_1_). The red dot indicates the maximum BF_10_ obtained (54.61 at r = 1.184), demonstrating that the result is robust to reasonable variations in the prior specification.


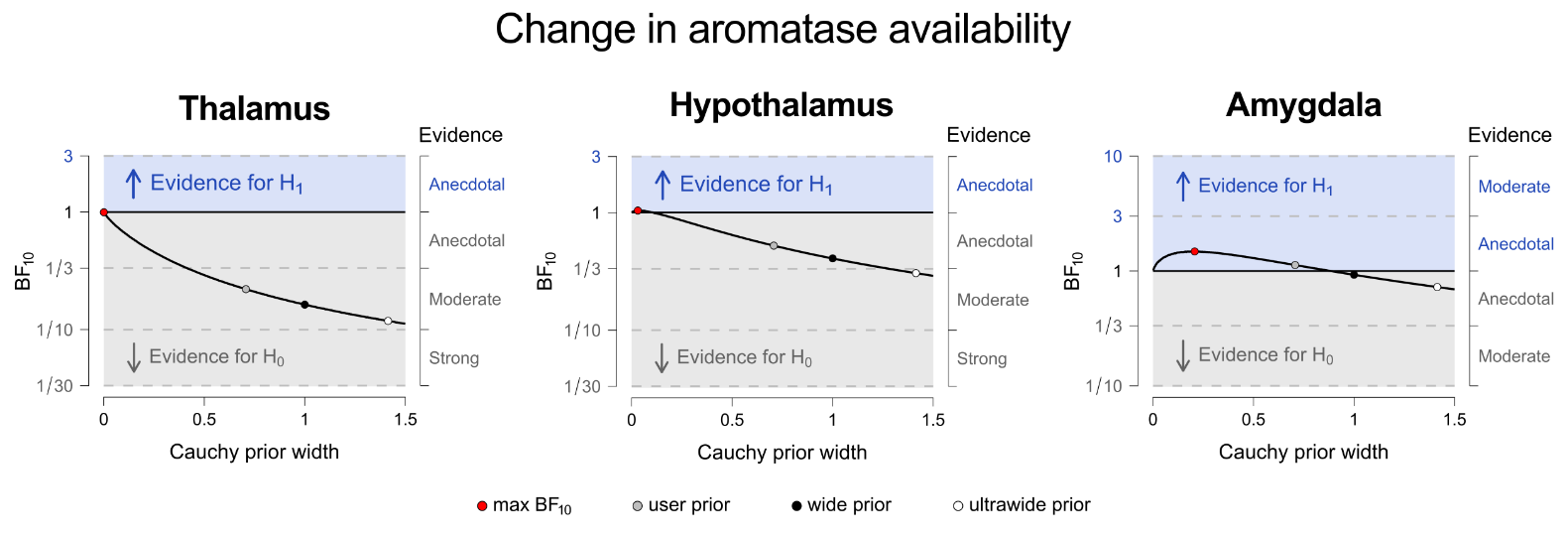


**Figure S4. Robustness check of the Bayes Factor for the effect of testosterone treatment on aromatase availability.** The robustness plots show how the Bayes Factor (BF_10_) for the increase in [^11^C]cetrozole binding changes with different Cauchy prior widths. The blue areas illustrate the evidence for the alternative hypothesis (H_1_), which posits that aromatase availability increases after one-week low-dose transdermal testosterone treatment. The grey areas illustrate the evidence for the null hypothesis (H_0_), which posits that aromatase availability does not change after one-week low-dose transdermal testosterone treatment. Across a wide range of priors (default user prior: r = 0.707; wide prior: r = 1; ultrawide prior: r = 1.414), the evidence supports H_0_, for the thalamus. BF_10_ values indicate that the strength of the evidence is moderate for the absence of changes in aromatase availability in the thalamus, weak (anecdotal) for the absence of changes in aromatase availability in the hypothalamus, and weak (anecdotal) for the increase in aromatase availability in the amygdala.

**
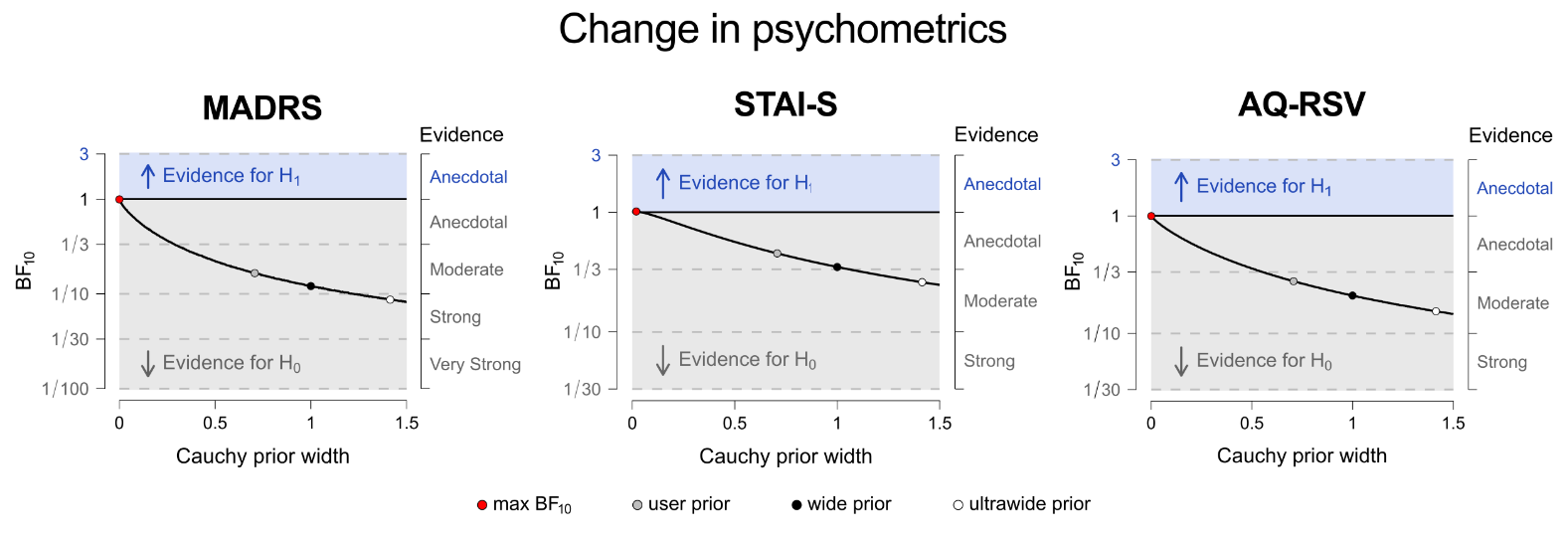
Figure S5. Robustness check of the Bayes Factor for the effect of testosterone treatment on psychometrics.** The robustness plots show how the Bayes Factor (BF_10_) for the decrease in depression (MADRS) and anxiety (STAI-S) scores, as well as for the increase in self-rated aggression (AQ-RSV), with different Cauchy prior widths. The blue areas illustrate the evidence for the alternative hypothesis (H_1_), which posits that changes in mood and behaviour are seen after one-week low-dose transdermal testosterone treatment, as described above. The grey areas illustrate the evidence for the null hypothesis (H_0_), which posits that psychometric scores do not change after one-week low-dose transdermal testosterone treatment. Across a wide range of priors (default user prior: r = 0.707; wide prior: r = 1; ultrawide prior: r = 1.414), the evidence supports H_0_ for depression and aggression scores. BF_10_ values indicate that the strength of the evidence is moderate for the absence of changes in MADRS and AQ-RSV scores, and weak (anecdotal) for the absence of changes in STAI-S scores.

|  | **Baseline**  **Median (IQR)** | **Post-treatment**  **mean ± SD** | **Difference** | |
| --- | --- | --- | --- | --- |
|  |  |  | **statistic (W)** | **p-value** |
| **Menstrual Cycle** |  |  |  |  |
| Cycle day | 6.5 (10.0) | 4.5 (23.0) | -0.24 | 0.81 |
| Oestradiol (pmol/L) | 132.32 (187.80) | 143.23 (159.68) | -0.15 | 0.88 |
| Progesterone (nmol/L) | 0.26 (17.06) | 0.55 (14.3) | 1.3 | 0.20 |

**Table S1. Menstrual cycle timing and hormonal characteristics across the baseline and post-treatment sessions.** Between-sessions differences in menstrual cycle day at scanning (based on forward counting), and serum gonadal hormones were assessed using paired-samples Wilcoxon signed rank tests in SPSS for Windows, version 30. Most participants were assessed in the early follicular phase of the menstrual cycle.

| **Spearman correlations** | **Post-treatment Thalamus BP_ND_** | **Post-treatment Hypothalamus BP_ND_** | **Post-treatment Amygdala BP_ND_** |
| --- | --- | --- | --- |
| **Post-treatment T** | *ρ* = 0.0, *p* = 1.0 | *ρ* = 0.2, *p* = 0.5 | *ρ* = -0.6, *p* = 0.1 |

**Table S2. Association between the post-treatment peripheral testosterone levels and the post-treatment oestrogen synthase availability.** Spearman rho (*ρ*) and *p* values are presented, illustrating the relationship between the serum testosterone concentrations (T) and the [^11^C]cetrozole binding (BP_ND_) following one-week of transdermal testosterone treatment.

| **Spearman correlations** | **Change in Thalamus BP_ND_** | **Change in Hypothalamus BP_ND_** | **Change in Amygdala BP_ND_** |
| --- | --- | --- | --- |
| **Change in T** | *ρ* = -0.2, *p* = 0.6 | *ρ* = 0.6, *p* = 0.1 | *ρ* = -0.1, *p* = 0.7 |

**Table S3. Association between the pre- to post-treatment change in peripheral testosterone levels and the change in oestrogen synthase availability.** Spearman rho (ρ) and *p* values are presented, illustrating the relationship between the change in serum testosterone concentrations (T) and the change in [^11^C]cetrozole binding (BP_ND_) from the pre-treatment to the post-treatment scanning session.

| **Spearman correlations** | **MADRS** | **STAI-S** | **AQ-RSV** |
| --- | --- | --- | --- |
| **Post-treatment T** | *r* = 0.0, *p* = 0.9 | *r* = -0.1, *p* = 0.7 | *r* = -0.1, *p* = 0.8 |

**Table S4. Association between the post-treatment peripheral testosterone levels and the post-treatment psychometrics.** Pearson coefficient (*r*) and *p* values are presented, illustrating the relationship between the serum testosterone concentrations (T) and the depression (MADRS), anxiety (STAI-S) and aggression (AQ-RSV) scores following one-week of transdermal testosterone treatment.

| **Spearman correlations** | **Change in MADRS** | **Change in STAI-S** | **Change in AQ-RSV** |
| --- | --- | --- | --- |
| **Change in T** | *r* = 0.44, *p* = 0.2 | *r* = 0.0, *p* = 0.9 | *r* = 0.6, *p* = 0.2 |

**Table S5. Association between the pre- to post-treatment change in peripheral testosterone levels and the change in psychometrics.** Pearson coefficient (*r*) and *p* values are presented, illustrating the relationship between the change in serum testosterone concentrations (T) and the change in depression (MADRS), anxiety (STAI-S) and aggression (AQ-RSV) scores from the pre-treatment to the post-treatment scanning session.
